# Discovering surface reaction pathways using accelerated molecular dynamics and network analysis tools†

**DOI:** 10.1039/d2ra04343b

**Published:** 2022-08-17

**Authors:** Hirotoshi Hirai, Ryosuke Jinnouchi

**Affiliations:** Toyota Central R&D Labs., Inc. 41-1, Yokomichi Nagakute Aichi 480-1192 Japan hirotoshih@mosk.tytlabs.co.jp

## Abstract

We present an automated method that maps surface reaction pathways with no experimental data and with minimal human interventions. In this method, bias potentials promoting surface reactions are applied to enable statistical samplings of the surface reaction within the timescale of *ab initio* molecular dynamics (MD) simulations, and elementary reactions are extracted automatically using an extension of the method constructed for gas- or liquid-phase reactions. By converting the extracted elementary reaction data to directed graph data, MD trajectories can be efficiently mapped onto reaction pathways using a network analysis tool. To demonstrate the power of the method, it was applied to the steam reforming of methane on the Rh(111) surface and to propane reforming on the Pt(111) and Pt_3_Sn(111) surfaces. We discover new energetically favorable pathways for both reactions and reproduce the experimentally-observed materials-dependence of the surface reaction activity and the selectivity for the propane reforming reactions.

## Abstract 2

We present an automated method that maps surface reaction pathways with no experimental data and with minimal human interventions.

## Graphical abstract

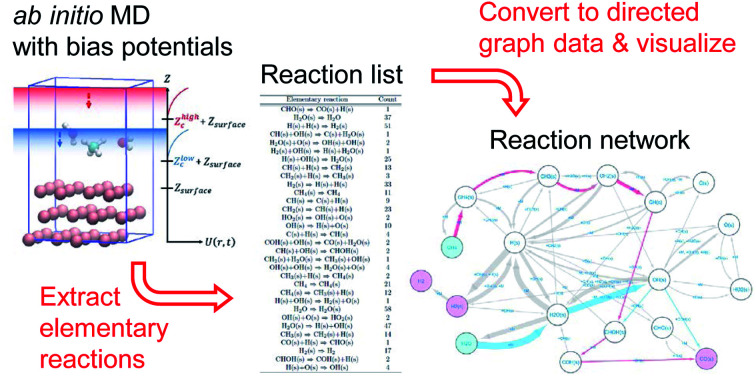

## 1 Introduction

Identification of surface reaction pathways is essential for mechanistic analysis of catalytic reactions and design of catalytic materials. Based on the identified reaction pathways, micro-kinetic reaction models^1^ can be constructed to quantitatively predict the reactivity and selectivity of the catalytic reactions under practical operation conditions of industrial reactors. *Ab initio* calculations are a vital tool for the identification of surface reaction pathways. Due to the significant advances in computational algorithms and computer performance,^2^ the reaction heats and activation energies of elementary reactions can now be predicted by *ab initio* calculations. The computed reaction heats and activation energies of a variety of surface elementary reactions exhibit universal linear relations^3–7^ that can be used to efficiently calculate reaction energies and activation energies of a wide variety of surface reactions. Micro-kinetic simulations using these thermodynamic and kinetic parameters have been successfully used to understand the mechanisms underlying the experimentally-observed material-dependent activity and selectivity trends and to design new catalysts with the desired performance.^8–10^

However, surface reactions are complex, and the number of the relevant chemical species and elementary reactions can become enormous, making it difficult to consider all possible reaction pathways. Therefore, surface reaction modeling studies have been conducted on the assumed reaction pathways that are suggested based on the experimental data and the researcher's intuition.^11,12^ Although reactants and products can be routinely detected in experiments, it is difficult to fully detect the reaction intermediates adsorbed on surfaces. Thus, experimental identification of reaction pathways is often challenging, and dominant reaction pathways may be overlooked.^13^ Needless to say, such empirical assumptions cannot be used for unknown reactive systems where experimental data are unavailable.

In the last 10 years, automatic reaction mechanism generation methods have been applied for computational predictions of surface reaction pathways. In particular, the power of automated methods was demonstrated in the studies of hydrocarbon combustion reactions.^14–16^ This success is due to the regularity of the skeleton of hydrocarbon molecules, where the additivity rule has been well-established, and the generation of the lists of elementary reactions and their rate constants can be automated based on the rule-based methods.^17^ For example, Zimmerman *et al.*^18,19^ proposed a method to generate reaction pathways using predefined heuristic bond-forming rules. Suleimanov and Green^20^ have proposed a fully automated method for reaction pathway identification where bond electron matrices were used to generate the possible pathways. Maeda *et al.*^21,22^ have developed an automated reaction pathway search method based on a geometric search. Habershon *et al.*^23,24^ have proposed an automated reaction mechanism generation method using Graph-driven search. Some of these methods have been extended to the surface reactions of hydrocarbon molecules and their oxides.^25–28^ These methods for the automatic generation of surface reaction pathways require the definition and validation of the rules for the generation of reaction networks. However, the definition and validation of such rules require a sufficiently large dataset and/or accumulated knowledge, and thus, are possible only for well-studied systems. This makes it difficult for such an approach to be applied to new systems.

Combinations of the automated methods with *ab initio* molecular dynamics (MD) simulations^29–31^ can provide a purely non-empirical procedure to identify reaction pathways. However, *ab initio* electronic structure computations are too computationally expensive to run MD simulations. In the previous studies, MD simulations using computationally low-cost quantum chemical calculation methods have been used to discover reactions, and high-precision calculations have been used to refine the discovered chemical reactions to obtain accurate reaction networks. For example, Wang *et al.*^32^ used Hartree-Fock level of theory with minimal basis set, and Varela *et al.*^33^ used a semi-empirical method for MD simulations to sample reactions. Nevertheless, it is still difficult to cover the required timescale of the chemical reactions, and therefore, direct *ab initio* MD identification of reaction pathways often necessitates unrealistically high simulation temperatures to accelerate the chemical reactions.^34^

To solve this problem, van Duin and others have developed the ReaxFF reactive force field that can describe bond formation and breaking while maintaining the speed of classical MD methods.^35^ In this approach, the force field parameters were determined to optimally reproduce *ab initio* data on selected relevant systems, and the constructed force field was used in MD simulations to observe the chemical reactions of interest. This approach has been successively applied to surface reaction simulations.^36–39^ However, the systems used to prepare the training dataset are often selected based on human intuitions, and therefore, the generated training dataset can lack information for important chemical reactions. It should also be noted that the limitations of the functional form of the force field can give rise to non-negligible errors.

An alternative method to solve this problem is to use accelerated MD methods. In these methods, biased potentials are introduced to accelerate the rate of rare events. After the simulations, thermodynamic and kinetic reaction properties of unbiased systems are restored by conversions based on statistical mechanics. Thus, chemical reactions can become observable within the timescale of the *ab initio* MD simulation. To date, a variety of accelerated MD methods have been proposed^40–44^ and applied to reactive systems.^45–49^ These methods have also been applied to surface systems, such as for the modeling of diffusion^50^ and heteroepitaxy reactions.^51^ However, to the best of our knowledge, this approach has never been applied to the catalytic reactions composed of a series of adsorption, bond scission, bond generation and desorption steps.

It should be noted that automated reaction identification method has not been established for the surface reactions. Several automated methods have been developed and applied to extract reaction pathways from MD trajectories in gas- and liquid-phase systems.^32,52,53^ However, no such method applicable to surface systems has been reported to date.

In this study, we present an automated method that can extract reaction pathways from the trajectories provided by accelerated *ab initio* MD simulations of catalytic reactions on heterogeneous surfaces. In this method, bias forces that promote surface reactions are applied to enable statistical sampling of the surface reactions. An automatic extraction of elementary reactions is realized by extending the method constructed for gas- or liquid-phase reactions.^49,54^ The extracted elementary reaction data are converted into directed graph data that can be efficiently mapped onto a clearly visible reaction network using a network analysis tool. To demonstrate its power, this method is applied to the steam reforming of methane on the Rh(111) surface and to propane reforming on the Pt(111) and Pt_3_Sn(111) surfaces. Steam reforming of methane (CH_4_ + H_2_O → CO + 3H_2_) on Rh(111) is a simple but important catalytic reaction that plays a key role in hydrogen production. Its energetics and reaction pathways have been intensively studied,^10,55,56^ so that the results obtained for this reaction can be verified by comparing to the *ab initio* computational results reported in previous studies. Pt_3_Sn(111) is known to have high selectivity for the propane to propylene (CH_3_CHCH_2_) conversion.^57^ Previous static *ab initio* calculations^58^ showed that the introduction of Sn activates propylene desorption, enhancing the catalytic selectivity of propane dehydrogenation. However, the propane dehydrogenation reaction involves a variety of elementary reactions that may not be fully examined by the static calculations. The application of our method to these systems demonstrate that our scheme can automatically extract energetically favorable reaction pathways from the accelerated *ab initio* MD simulations without using any empirical information.

## 2 Method

### 2.1 Accelerated MD simulation for surface reaction

In this study, slab models were used to represent the gas/solid interface. In the slab models, periodic boundary conditions are imposed on the unit cells in order to accurately represent the infinite extent of the single crystal surfaces, where atoms are repeatedly arranged in the surface horizontal directions, and a vacuum layer is inserted between the surface slabs to represent the boundary between the gas phase and the solid surface. The dimensions of the slab model are specified by the number of primitive surface unit cells included in the supercell, the number of slab layers and the thickness of the vacuum layer are chosen so that the model can be used to accurately provide the desired properties. For example, a thicker slab is necessary to accurately describe the interactions of the molecules with bulk surfaces, and a thicker vacuum layer is necessary to avoid artificial interactions with the molecules and slabs in the periodic image cells. A wider unit cell is required to examine adsorbate energetics at low coverage. For molecular dynamics simulations, additional care is also required to prevent the artificial interactions between the periodic images of the slabs. In the simulations, the molecules placed in the gas phase can interact with surfaces located at both upper and lower boundaries of the gas phase even though in reality, the molecule can interact only with a surface at one boundary. Additionally, it is also necessary to ensure efficient sampling. If a thick vacuum layer is placed between the slabs, the molecule can spend most of the simulation time in that layer, making the sampling of the desired interfacial reactions significantly worse.

**Fig. 1:**
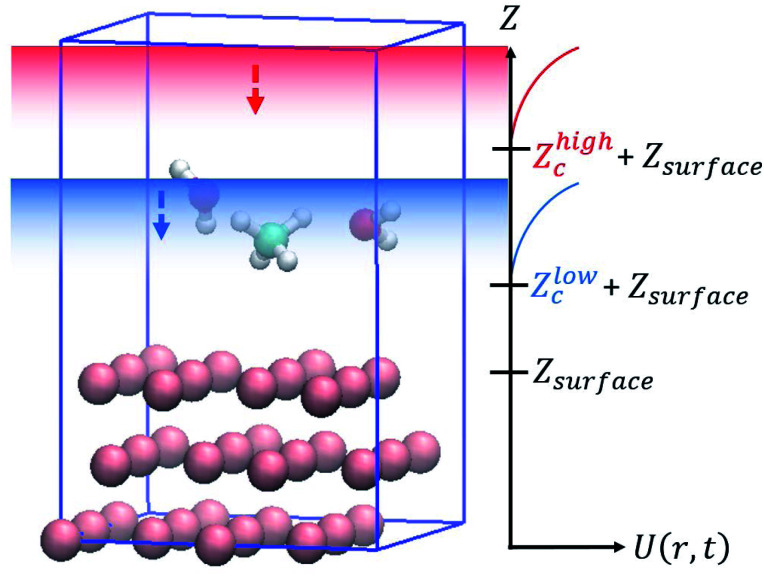
Boundary conditions in the accelerated molecular dynamics simulations for surface reactions.

To solve these problems, in this work, wall potentials are imposed to prevent the molecular diffusion to the vacuum layer using the following expression:1
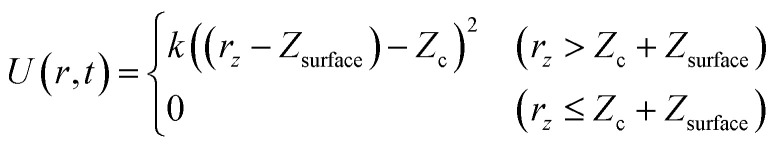
where *r*_*z*_ is the *Z* coordinate (surface vertical direction) of each atom, *Z*_surface_ is the position of the top layer of the slab, and *k* and *Z*_c_ are parameters that control the strength and position of the wall potentials. As shown in Fig. 1, *U*(*r*, *t*) was applied for each of the two *Z*_c_: *Z*^high^_c_, *Z*^low^_c_.

Both wall potentials apply a spring force to the atoms located at *z* > *Z*_c_ + *Z*_surface_ to ensure that they return to the surface. Since the wall potentials do not act on the atoms located at *z* ≤ *Z*_c_ + *Z*_surface_, they do not affect the energetics of surface reactions. The wall potential located at *Z*^high^_c_ acts on atoms continuously to prevent molecular diffusion to the upper boundary of the vacuum layer. By contrast, the wall potential located at *Z*^low^_c_ is periodically turned on and off at constant intervals. When this wall potential is turned on, it promotes the adsorption of the molecule on the surface. The application of this bias force temporarily increases the velocity of the gas phase molecule moving toward the surface, increasing its temperature. This enables efficient sampling of the reactions by the molecules distributed in the high-energy region of the Boltzmann distribution.

In addition to the application of wall potentials (eqn (1)), the accelerated MD method was used to enhance the sampling of surface reactions. We used the adaptive hyperdynamics (AHD) method^43^ to accelerate the reactions. The AHD method is based on the hyperdynamics method developed by Voter.^40^ Voter showed that the relative rates of transitions from one state to other states are preserved as long as the original potentials at the transition states are not modified by the bias potential.^40^ This means that the selectivity of the reaction pathways are preserved if the bias potential is designed so as not to perturb the potential energy surfaces near the transition states. Such a bias potential was proposed by Hamelberg *et al.*^41^ as shown below.2
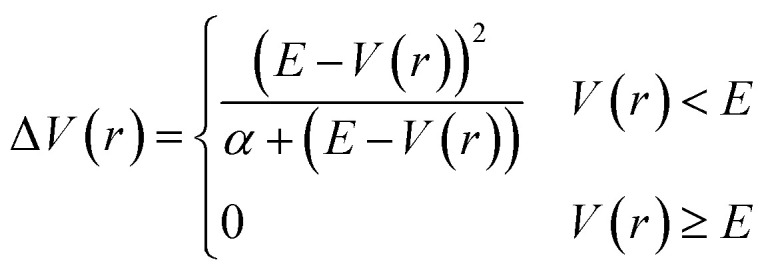
where *V*(*r*) is the potential energy of the system, *α* is a parameter that modulates the depth of the modified potential, and *E* is a constant parameter in the original scheme. In AHD simulations, *E* was adaptively controlled to accelerate rare events. In these schemes, the parameter *E* is updated at every time interval *τ* according to3
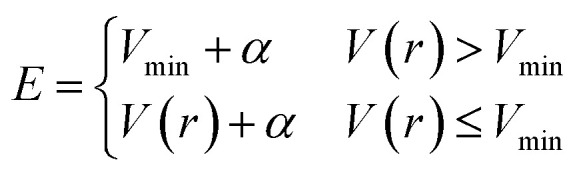
where *V*_min_ is updated to be the minimum potential energy in the previous interval. This scheme is also used in the present study. The details of the AHD method and examples of its application can be found in ref. 43, 48, 49 and 59.

The AHD method was implemented in the Quantum Espresso *ab initio* simulation package.^60^ The parameters of the AHD were set to *α* = 0.13 Ry and *τ* = 100 MD steps. For the parameters in eqn (1), *k* = 0.008 Ry Å^−2^, *Z*^low^_c_ = 2 Å, and *Z*^high^_c_ = 7.5 Å were used. The wall potential corresponding to *Z*^low^_c_ was switched on and off every 250 MD steps. Because of the limited width of the surface unit cell, hydrogen atoms dissociated from the molecules are repeatedly adsorbed on and desorbed from the surface during AHD simulations. These frequent phenomena strongly hinder efficient sampling of other rare events. To prevent repeated hydrogen adsorptions and desorptions, the bias potential located at *Z*^low^_c_ was always switched off for the hydrogen molecules desorbed from the surface. This enables the hydrogen molecules generated by the reactions to diffuse to the vacuum region. Additionally, we deleted the hydrogen molecules that diffuse to the vacuum region.

### 2.2 Automatic extraction of elementary reactions and visualization of reaction networks

In this study, we assumed that metal atoms in the slab do not evaporate and travel to the gas phase. We also assumed that subsurface oxides are not formed and that the crystal structure of the metal slab is preserved during AHD simulations. Hence, we focus only on the reactions on clean surfaces. To automatically extract elementary reactions, the automatic elementary reaction extraction method^49,54^ that was developed for gas- and liquid-phase reactions was applied to the group of atoms comprising the reactant molecules located in the gas phase at the beginning of the simulations. In this method, a bond matrix that identifies each atomic pair as either bonding or non-bonding using 1 or 0, respectively, is block-diagonalized to extract the chemical species and elementary reactions from the MD trajectories (see ESI for details†). The bond matrix was updated every 5 MD steps. To distinguish between the surface and gas-phase chemical species, the distances between the atoms located in the gas phase at the beginning of the simulations and the atoms in the slab were calculated. When the distances became shorter than the determined threshold value, these atoms were assigned as surface species and labeled with “(s)”. This procedure was conducted after every evaluation of the bond matrix. Then, we can detect adsorption or desorption reactions based on the changes in these labels. The following threshold values were used to determine whether the atom is assigned to the surface-adsorbed species (Table 1).

**Table 1:** Thresholds used to determine surface adsorbed species

Atom pair	Threshold (Å)
H–Rh	2.40
O–Rh	2.76
C–Rh	2.76
H–Pt	2.40
C–Pt	2.76
H–Sn	2.40
C–Sn	2.76

The extracted elementary reactions were mapped onto a reaction network using network graph analysis tools that can clearly visualize the reaction pathways and numbers of events. This visualization process was automated using Python with NetworkX modules.^61^ The constructed graph data were written to a file in the graph modeling language (GML) format and were visualized by the Cytoscape software.^62,63^

### 2.3 Models and parameters

**Fig. 2:**
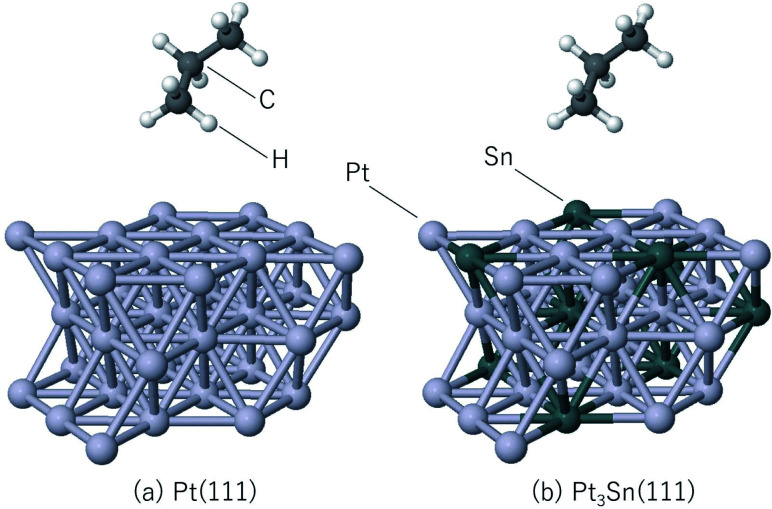
Slab models for (a) Pt(111), (b) Pt_3_Sn(111).

The steam reforming of methane on the Pt(111) surface was simulated using a slab model of 12 atoms with the
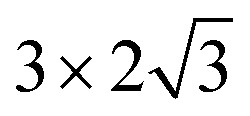
 periodicity and with the thickness of 3 atomic layers (see Fig. 1). The atoms in the bottom layer of the slab were fixed at the theoretically calculated positions for the bulk Rh. The vacuum layer was set to 12 Å. A methane molecule and two water molecules acting as reactant molecules were located in the vacuum layer at the beginning of the simulations. Propane dehydrogenation on the Pt_3_Sn alloy surface was simulated using a three-layer slab model with the 
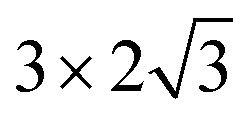
 periodicity (Fig. 2).

The thickness of the vacuum layer was set to 12 Å. The structure of Pt_3_Sn was constructed according to the previous work.^58^ A propane molecule was placed in the gas phase as the initial state to simulate the propane reforming reaction. During the simulations, the atoms in the bottom layer were fixed to the bulk positions obtained in the bulk calculations. All simulations were carried out using the Quantum Espresso^60^*ab initio* simulation package that uses the plane wave basis set and pseudopotentials. Vanderbilt ultrasoft pseudopotentials^64^ were used to describe the electron–ion interaction. The cutoff energy of the plane wave basis set was set to 30 Ry and revPBE^65–67^ was used as the exchange-correlation energy functional. The time step of the MD calculation was set to 15 Ry a.u. (0.726 fs), and 5000 step MD simulations were conducted 10 times for the steam reforming of methane, 20 times for the propane reforming simulations with each simulation started from different initial positions and velocities in order to sample the reactions. The temperature was set to 1273 K, and the Berendsen thermostat^68^ was used for temperature control during the MD simulations. Gaussian smearing with the energy width of 0.003 Ry was used to improve the convergence of the electronic structure calculations.^69^

## 3 Results and discussion

### 3.1 Methane steam reforming

A total 32 types of elementary reactions were observed during the 10 independent AHD simulations. The number of events for each elementary reaction is tabulated in Table 2.

**Table 2:** Extracted elementary reactions for methane steam reforming on Rh(111)

Elementary reaction	Count
CHO(s) ⇒ CO(s) + H(s)	1
H_2_O(s) ⇒ H_2_O	37
H(s) + H(s) ⇒ H_2_(s)	51
CH(s) + OH(s) ⇒ C(s) + H_2_O(s)	1
H_2_O(s) + O(s) ⇒ OH(s) + OH(s)	2
H_2_(s) + OH(s) ⇒ H(s) + H_2_O(s)	1
H(s) + OH(s) ⇒ H_2_O(s)	25
CH(s) + H(s) ⇒ CH_2_(s)	13
CH_2_(s) + H(s) ⇒ CH_3_(s)	3
H_2_(s) ⇒ H(s) + H(s)	33
CH_4_(s) ⇒ CH_4_	11
CH(s) ⇒ C(s) + H(s)	9
CH_2_(s) ⇒ CH(s) + H(s)	23
HO_2_(s) ⇒ OH(s) + O(s)	2
OH(s) ⇒ H(s) + O(s)	10
C(s) + H(s) ⇒ CH(s)	4
COH(s) + OH(s) ⇒ CO(s) + H_2_O(s)	2
CH(s) + OH(s) ⇒ CHOH(s)	2
CH_2_(s) + H_2_O(s) ⇒ CH_3_(s) + OH(s)	1
OH(s) + OH(s) ⇒ H_2_O(s) + O(s)	4
CH_3_(s) + H(s) ⇒ CH_4_(s)	2
CH_4_ ⇒ CH_4_(s)	21
CH_4_(s) ⇒ CH_3_(s) + H(s)	12
H(s) + OH(s) ⇒ H_2_(s) + O(s)	1
H_2_O ⇒ H_2_O(s)	58
OH(s) + O(s) ⇒ HO_2_(s)	2
H_2_O(s) ⇒ H(s) + OH(s)	47
CH_3_(s) ⇒ CH_2_(s) + H(s)	14
CO(s) + H(s) ⇒ CHO(s)	1
H_2_(s) ⇒ H_2_	17
CHOH(s) ⇒ COH(s) + H(s)	2
H(s) + O(s) ⇒ OH(s)	4

Formation of CO(s) (CO molecule adsorbed on the surface) was observed twice during the simulations while its desorption from the surface was not observed because of the high adsorption energy(1.3 eV) of CO on the Rh(111) surface. By contrast, formation of H_2_(s) (H_2_ molecule adsorbed on the surface) through H(s) + H(s) → H_2_(s) was observed 51 times, and its desorption from the surface was observed 17 times. Thus, the steam reforming of methane to CO and H_2_ was simulated by our accelerated MD simulations.

**Fig. 3:**
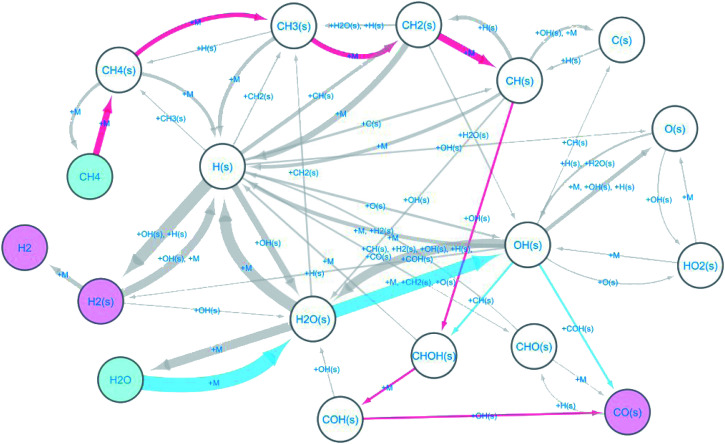
Reaction network for methane steam reforming on Rh(111).

The observed elementary reactions were converted to a directed graph data and were summarized as a reaction network with 18 nodes and 53 edges shown in Fig. 3.

Here, the label “+ M” means an unimolecular reaction. The pathways leading to the formation of CO(s) from CH_4_ and H_2_O are colored red and blue, respectively. The reaction network indicates that at the beginning of the reforming, methane and water are decomposed on the surface by the following reactions.

R1 CH_4_ ⇒ CH_4_(s)

R2 CH_4_(s) ⇒ CH_3_(s) + H(s)

R3 CH_3_(s) ⇒ CH_2_(s) + H(s)

R4 CH_2_(s) ⇒ CH(s) + H(s)

R5 H_2_O ⇒ H_2_O(s)

R6 H_2_O(s) ⇒ OH(s) + H(s)

The formed surface-adsorbed hydrogen atoms quickly associate on the surface to form the hydrogen molecule.

R7 H(s) + H(s) ⇒ H_2_(s) ⇒ H_2_

The slow CO formation reaction occurs through the following surface reactions.

R8 CH(s) + OH(s) ⇒ CHOH(s)

R9 CHOH(s) ⇒ COH(s) + H(s)

R10 COH(s) + OH(s) ⇒ CO(s) + H_2_O(s)

**Fig. 4:**
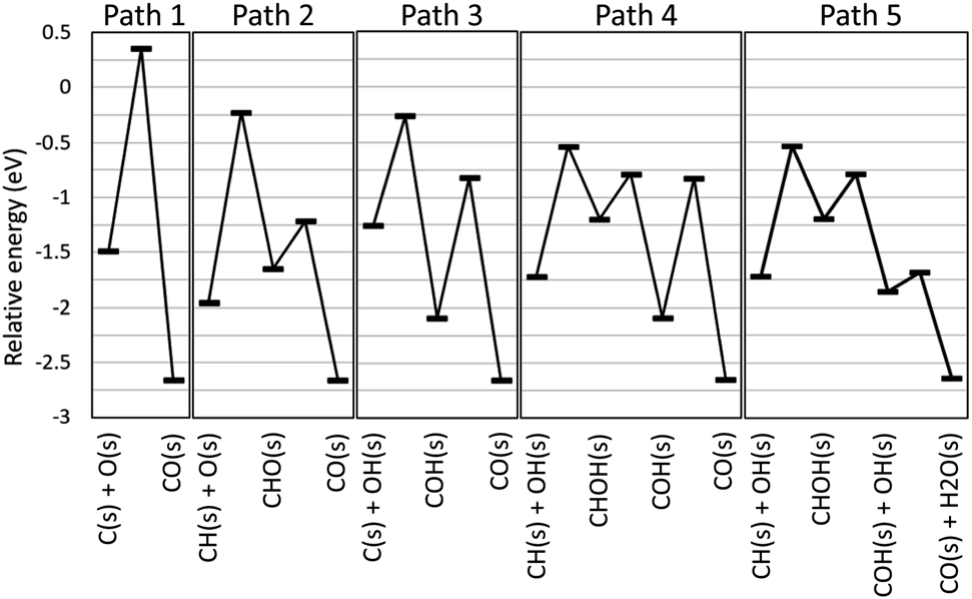
Energy diagram for methane steam reforming on Rh(111).

The automatically extracted CO formation pathways R8 and R9 agree with the energetically most favorable pathway suggested by elaborate manual static *ab initio* calculations.^70^ However, it should be noted that to the best of our knowledge, reaction path R10 has never been considered in previous work. To verify this newly discovered pathway, we examined its energetics by static *ab initio* calculations using the Climbing Image-Nudged Elastic Band (CI-NEB) method^71^ and compared its energetics with those of other pathways as shown in Fig. 4.

Here, the reference energy value was set to the energy of one methane and two water molecules in the gas phase, and all species not shown were assumed to be adsorbed on the surface as H(s) and O(s). Paths 1, 2, and 3 were included in the existing reaction models for the methane steam reforming on Rh surface.^56^ However, they exhibit high energies for the transition states of C(s) + O(s) ⇒ CO(s) for Path 1, CH(s) + O(s) ⇒ CHO(s) for Path 2 and C(s) + OH(s) ⇒ COH(s) for Path 3, indicating that these pathways are kinetically hindered. As suggested by Lee and co-workers,^70^ Path 4 exhibits the lower transition state of CH(s) + OH(s) ⇒ CHOH(s). Following this step, COH(s) is formed from CHOH(s). Identical steps appear in our Path 5, however, Paths 4 and 5 differ in the subsequent CO formation step. In Path 4, the unimolecular decomposition reaction of COH(s) occurs to form CO(s) and H(s). This step has a large activation energy of 1.27 eV. By contrast, in Path 5, the hydrogen transfer reaction from COH(s) to OH(s) occurs to form CO(s) and H_2_O(s). Remarkably, this hydrogen transfer reaction is barrierless while the CI-NEB analysis finds a slight energetic barrier in the subsequent OH(s) diffusion process. Although formation of OH(s) involves activation barriers of 1.15 eV for H_2_O(s) ⇒ H(s) + OH(H_3_CH_2_CH_2_)(s) ⇒ CH_3_CHCH_2_(s) and of 0.87 eV for O(s) + H(s) ⇒ OH(s), both of these are lower than the activation barrier of the unimolecular decomposition reaction (1.27 eV). Hence, static *ab initio* calculations indicate that Path 5 discovered automatically by our scheme is indeed an energetically more favorable pathway than the pathways reported in previous studies.

### 3.2 Propane reforming (dehydrogenation) on Pt(111) and Pt_3_Sn(111) surfaces

**Fig. 5:**
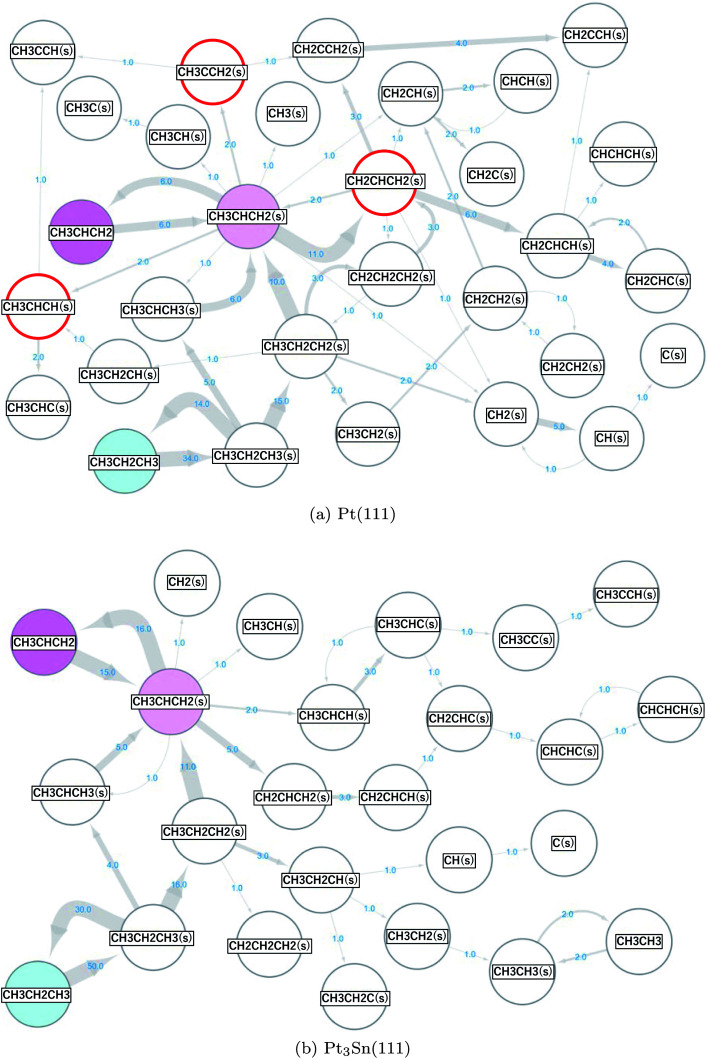
Propane reaction networks obtained on the (a) Pt(111) and (b) Pt_3_Sn(111) surfaces.

The same scheme was applied to the propane (CH_3_CH_2_CH_3_) reforming reaction on the Pt(111) and Pt_3_Sn(111) surfaces to elucidate the origin of the high selectivity of the Pt_3_Sn(111) surface for propylene (CH_3_CHCH_2_) production from propane (CH_3_CH_2_CH_3_).^57,58^ A reaction network with 34 nodes and 77 edges was obtained for Pt(111), and a reaction network with 28 nodes and 53 edges was obtained for Pt_3_Sn(111). These networks are shown in Fig. 5, where the surface hydrogen atoms (H(s)) are not shown to simplify the networks. The propane in the gas phase (the initial state) is shown in cyan, the propylene in the gas phase (the main target of the reforming reaction) is shown in magenta, and the propylene adsorbed on the surface (CH_3_CHCH_2_(s)) is shown in light magenta. The numbers on the arrows indicate the numbers of the occurrences of the corresponding events. More frequent events are shown by thicker arrows. More nodes, edges and arrows appear in the network for the reaction on the Pt(111) than in the network for the reaction on the Pt_3_Sn(111) surface, indicating that the Pt(111) surface is more reactive than the Pt_3_Sn(111) surface. On the Pt(111) surface, propane is decomposed to propylene and is further decomposed to CH_2_CHCH_2_(s), CH_2_CHCH(s), and CH_2_CHC(s) through the following reactions.

R11 CH_3_CH_2_CH_3_ ⇒ CH_3_CH_2_CH_3_(s)

R12 CH_3_CH_2_CH_3_(s) ⇒ CH_3_CH_2_CH_2_(s) + H(s)

R13 CH_3_CH_2_CH_2_(s) ⇒ CH_3_CHCH_2_(s) + H(s).

R14 CH_3_CHCH_2_(s) ⇒ CH_2_CHCH_2_(s) + H(s)

R15 CH_2_CHCH_2_(s) ⇒ CH_2_CHCH(s)+H(s)

On the other hand, the formed propylene is desorbed to the gas phase on the Pt_3_Sn(111) surface.

R16 CH_3_CHCH_2_(s) ⇒ CH_3_CHCH_2_

The results are consistent with the experimentally observed low reactivity and high selectivity for selective propylene formation on the PtSn alloy catalyst.^57^

Fig. 5 shows the presence of a side pathway for the propylene formation on both the Pt(111) and Pt3Sn(111) surfaces.

R17 CH_3_CH_2_CH_3_(s) ⇒ CH_3_CHCH_3_(s) + H(s)

R18 CH_3_CHCH_3_(s) ⇒ CH_3_CHCH_2_(s) + H(s)

As indicated by the thickness of the arrows, pathways R12 and R13 occur more frequently than pathways R17 and R18. Static *ab initio* CI-NEB calculations support the observations in the AHD simulations as shown by the data presented in Table 3.

**Table 3:** Activation energies of propane dehydrogenation reactions on the Pt(111) and Pt_3_Sn(111) surfaces^a^

Elementary reaction	Activation energy (eV)	
	Pt(111)	Pt_3_Sn(111)
⇒ CH_3_CH_2_CH_2_(s) + H(s)	0.69	0.75
⇒ CH_3_CHCH_3_(s) + H(s)	0.70	0.78

a On both Pt(111) and Pt_3_Sn(111) surfaces, pathways R12 and R13 through CH_3_CH_2_CH_2_(s) have lower activation barriers than pathways R17 and R18 through CH_3_CHCH_3_(s).

On the Pt(111) surface, selective formation of propylene is hindered by further dehydrogenation reactions. The decomposition to CH_2_CHCH_2_(s) was observed most frequently (11 times), followed by the decomposition to CH_3_CCH_2_(s) and decomposition to CH_3_CHCH(s) that were observed twice each in this study (these 3 products are highlighted by red circles in Fig. 5(a)). These results are different from the conclusions of the previous studies^58,72^ that reported that the decompositions to CH_3_CCH_2_(s) and CH_3_CHCH(s) rather than to CH_2_CHCH_2_(s) are the main pathways. To verify our results, we further conducted CI-NEB analyses. The obtained results are summarized in Table 4.

**Table 4:** Activation energies and reaction heats for the propylene dehydrogenation reactions on the Pt(111) surface. Literature values are given in parentheses^72^

Elementary reaction	Activation energy (eV)	Reaction heat (eV)
⇒ CH_2_CHCH_2_(s) + H(s)	0.756	−0.003
⇒ CH_3_CCH_2_(s) + H(s)	0.846 (0.77)	−0.001 (−0.01)
⇒ CH_3_CHCH(s) + H(s)	0.810 (0.76)	0.007 (0.06)

The computed activation barriers for the reactions for the formation of CH_3_CCH_2_(s) and CH_3_CHCH(s) suggested in previous work are in good agreement with the reported values albeit with slight differences because of the differences in the computational methods (this study: revPBE/ultrasoft pseudopotentials, the previous study: PBE/projector augmented wave method). The activation barrier of the CH_2_CHCH_2_(s) formation reaction is the lowest among the three reactions. Hence, our scheme correctly automatically extracts the energetically favorable pathway.

## 4 Conclusion

An automated *ab initio* method was developed to elucidate the reaction pathways of complex surface reactions with no experimental data and with minimal human interventions. The method adopts accelerated MD simulations that enable efficient sampling of rare events at the surface by applying bias potentials without perturbing the selectivity of multiple elementary reactions. By extending the method previously developed for gas- or liquid-phase reactions, elementary reactions are automatically extracted from the MD trajectories. The extracted elementary reactions are converted to directed graph data that can be visualized by a network analysis tool. The method was applied to two surface reactions: the steam reforming of methane on the Rh(111) surface and propane dehydrogenation on the Pt(111) and Pt_3_Sn(111) surfaces. In both applications, our automated scheme extracted new energetically favorable reaction pathways as well as conventional reaction pathways suggested in past studies that employed manual static *ab initio* calculations. Moreover, the application of our method to the propane dehydrogenation showed that the high selectivity for propylene formation on the Pt_3_Sn(111) surface is due to the deactivation of propylene decomposition and the activation of propylene desorption on this surface. These two applications demonstrate that our scheme can indeed automatically extract the dominant reaction pathways of complex heterogeneous surface reactions without making artificial assumptions regarding the reaction pathways.

## Conflicts of interest

There are no conflicts to declare.

## Supplementary Material

RA-012-D2RA04343B-s001
