# Supplementary material for: Discovering surface reaction pathways using accelerated molecular dynamics and network analysis tools
Source: RSC Adv. 2022 Aug 17;12(36):23274–83. doi: 10.1039/d2ra04343b (PMC9382359; doi:10.1039/d2ra04343b)
Supplement: RA-012-D2RA04343B-s001 [file RA-012-D2RA04343B-s001.pdf]

Electronic Supplementary Material (ESI) for RSC Advances.
This journal is © The Royal Society of Chemistry 2022

Supporting Information for "Discovering Surface Reaction Pathways Using Accelerated Molecular Dynamics and Network Analysis Tools"

Hirotoshi Hirai* and Ryosuke Jinnouchi

Toyota Central R&D Labs., Inc., 41-1, Yokomichi, Nagakute, Aichi 480-1192, Japan

I. SAMPLING ELEMENTARY REACTIONS

Here, we describe a method to extract elementary reactions from the trajectories of molecular dynamics (MD) simulations.

A. Identification of chemical species

At first, we describe a method to extract the chemical species contained in a system from the atomic coordinates obtained by MD simulations. For each atomic pair, bonding or no bonding was described using a "bond matrix",

$$M[i,j] = \begin{cases} 1 \text{ (bonding)} \\ 0 \text{ (no bonding)} \end{cases} \tag{1}$$

where i and j are the labels specifying atoms (here, atoms composing the slab are excluded). This matrix was updated every 5 steps of the MD simulation. In this study, bonding or no bonding was determined from the inter-atomic distance; if the distance is shorter than the cutoff radius, the pair of atoms is judged to form a bond. Different cutoff parameters were used to detect bond creations ($M[i,j] : 0 \rightarrow 1$) and bond breakings ($M[i,j] : 1 \rightarrow 0$). Shorter cutoff parameters were used for bond creations, and longer cutoff parameters were used for bond breakings to avoid wrong assignments of molecular vibrations (bond stretching and contraction) and non-reactive collisions (temporary reduction of inter-atomic distance)

TABLE I. Bond cutoff parameters

	M:0→1	M:1→0
H-H	0.814	1.894
C-C	1.694	2.464
O-O	1.628	2.368
H-C	1.210	1.760
H-O	1.100	1.600
C-O	1.452	2.112

* hirotoshih@mosk.tytlabs.co.jp

to chemical reactions. The bond cutoff parameters used in this study are summarized in TABLE I.

Isolated atomic groups can be identified by the block-diagonalization of the bond matrix. The block-diagonalization can be executed by permutations of rows and columns of the bond matrix. This process is schematically shown in FIG. 1. An isolated atomic group can be found in each block in the block-diagonalized matrix, and the identified atomic group corresponds to the isolated molecule. In this study, a brute-force method was used

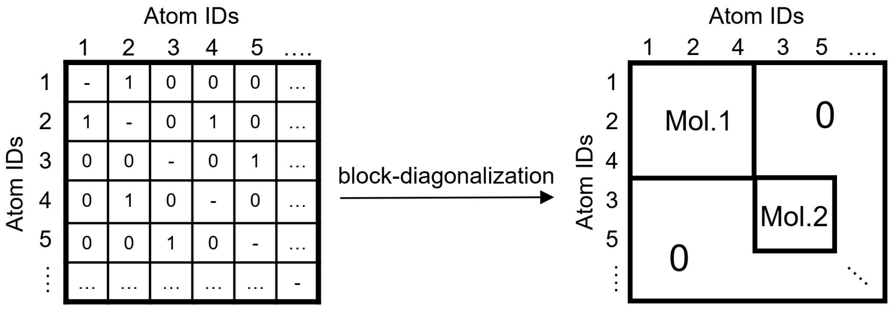

FIG. 1. Partitioning of a group of atoms into molecules by block diagonalization of the bond matrix.

for the block-diagonalization. The composition formula ($C_xH_yO_z$) of each molecule can be constructed from the atoms that constitute each block. However, if isomers present, the composition formula is not sufficient. In such a case, we can use SMILES format to represent molecules in text lines. The molecular structure file like XYZ format can be constructed for each molecule from the MD trajectory data and atomic IDs. The molecular structure files can be converted to SMILES format using OpenBabel software, for example.

In this manner, the type and number of chemical species present in the system at every MD step can be determined.

B. Identification of chemical reactions

Here, we describe a method to extract elementary reactions from the trajectories of molecular dynamics simulations. Changes in the bond matrix during the MD simulations mean changes in the bonds between atoms caused by chemical reactions. Therefore, chemical reactions can be extracted by detecting the time variation of the bond matrix.

Let M^t be the bond matrix at t-th MD step, and assume that M^t is the matrix, whose rows and columns are arranged in the atomic ID order before the block diagonalization as shown in FIG. 1. Here, we compare M^t and M^{t-1}. If there are differences in the matrix elements between M^t and M^{t-1}, atoms corresponding to these matrix elements are judged to be involved in chemical reactions.

It should be, however, noted that strong molecular vibrations and inter-molecular collisions at high temperatures can result in temporary changes in the bond matrix, and those changes can be wrongly assigned to chemical reactions even though we try to prevent them by using two different cutoff values. To avoid the wrong assignments, not only M^t and M^{t-1} but also M^{t+1} and M^{t-2} were used to judge whether chemical reactions happen or not. Even if there is a difference between M^t and M^{t-1}, if $M^{t-2} = M^t$ or $M^{t-1} = M^{t+1}$, the change from M^{t-1} to M^t is judged to just a temporal change and is not assigned to a reaction. The use of the history of the bond matrix in this way can drastically reduce the probability of the wrong assignments.

When the change in the bond matrix is assigned to a chemical reaction, the molecules involved to the reaction can be determined from the atomic IDs that exhibit the changes in the matrix elements. The molecules involved in the reaction obtained from M^{t-1} are assigned to the reactants, for example, A and B, and those obtained from M^t are assigned to the products, C and D. By using the identified species, the reaction equation can be output as A + B → C + D. As in this example, multiple molecules can be involved in the identified chemical reaction. Without any sorting of the chemical species, the reaction, A + B → C + D and B + A → C + D (or A + B → D + C and so on) can be judged to be different reaction although they are actually identical. To prevent this, the extracted molecules are sorted according to a predetermined procedure. In this study, the molecules were sorted according to their molecular names (composition formula).

It should be also noted that multiple elementary reactions can simultaneously happen and that the reaction equation extracted in this procedure may not be necessarily an elementary reaction equation. The multiple complex reaction can be split to elementary reactions by the following manner. Here, we consider the case where a complex reaction equation, A + B + C → D + E + F + G, is extracted, where A to G are arbitrary molecular species. If an atomic ID in the reactant molecule A is, for example, involved also in the product molecule D, then A and D molecules must be included in an elementary reaction equation. By examining

the relationships of the atomic IDs in the reactants and products, for example, as shown in FIG. 2, the complex reaction can be split to the elementary reactions: A + B → D + E and C → F + G. This procedure is equivalent to the blocking of a matrix representing the relationships of the involvement of the atomic IDs as illustrated in FIG. 3. Here, when the reactant A and product D own the same atomic ID, the matrix element between A and D is set to 1. Otherwise, the element is set to 0. Blocking the matrix provides the matrix shown in FIG. 3, and the each block in this matrix provides the reactants and products involved in one elementary reaction. The blocking was executed also by a brute-force method.

By this way, elementary reactions can be automatically extracted from MD trajectories.

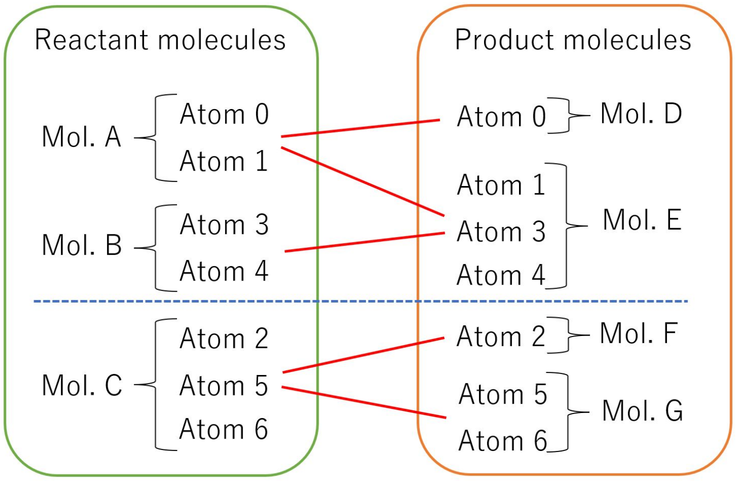

FIG. 2. Partitioning of the apparent many-body reaction into elementary reactions.

Product molecules

Reactant molecules	Mol. D	Mol. E	Mol. F	Mol. G
Mol. A	1	1	0	0
Mol. B	0	1	0	0
Mol. C	0	0	1	1

FIG. 3. Partitioning of the apparent many-body reaction into elementary reactions: matrix representation.
